# A Robust GPS Navigation Filter Based on Maximum Correntropy Criterion with Adaptive Kernel Bandwidth

**DOI:** 10.3390/s23239386

**Published:** 2023-11-24

**Authors:** Dah-Jing Jwo, Yi-Ling Chen, Ta-Shun Cho, Amita Biswal

**Affiliations:** 1 Department of Communications, Navigation and Control Engineering, National Taiwan Ocean University, 2 Peining Rd., Keelung 202301, Taiwan; ylchen662488@gmail.com (Y.-L.C.); amitabiswal1988@gmail.com (A.B.); 2 Department of Business Administration, Asia University, 500 Liufeng Road, Wufeng, Taichung 41354, Taiwan; cho2022@asia.edu.tw

**Keywords:** Gaussian noise, heavy-tailed noise, extended Kalman filter, GPS, maximum correntropy criterion, adaptive kernel bandwidth

## Abstract

Multiple forms of interference and noise that impact the receiver’s capacity to receive and interpret satellite signals, and consequently the preciseness of positioning and navigation, may be present during the processing of Global Positioning System (GPS) navigation. The non-Gaussian noise predominates in the signal owing to the fluctuating character of both natural and artificial electromagnetic interference, and the algorithm based on the minimum mean-square error (MMSE) criterion performs well when assuming Gaussian noise, but drops when assuming non-Gaussian noise. The maximum correntropy criteria (MCC) adaptive filtering technique efficiently reduces pulse noise and has adequate performance in heavy-tailed noise, which addresses the issue of filter performance caused by the presence of non-Gaussian or heavy-tailed unusual noise values in the localizing measurement noise. The adaptive kernel bandwidth (AKB) technique employed in this paper applies the calculated adaptive variables to generate the kernel function matrix, in which the adaptive factor can modify the size of the kernel width across a reasonably appropriate spectrum, substituting the fixed kernel width for the conventional MCC to enhance the performance. The conventional maximum correntropy criterion-based extended Kalman filter (MCCEKF) algorithm’s performance is significantly impacted by the value of the kernel width, and there are certain predetermined conditions in the selection based on experience. The MCCEKF with a fixed adaptive kernel bandwidth (MCCEKF-AKB) has several advantages due to its novel concept and computational simplicity, and gives a qualitative solution for the study of random structures for generalized noise. Additionally, it can effectively achieve the robust state estimation of outliers with anomalous values while guaranteeing the accuracy of the filtering.

## 1. Introduction

Due to multipath interference and non-line-of-sight (NLOS) reception, global navigation satellite systems (GNSS), such as the Global Positioning System (GPS), generate significant errors in urban settings [1,2]. Due to diffraction from adjacent structures like the ground and water surfaces, structures, moving cars, hills, trees, etc., and multipath effects arise when GPS signals travel via many pathways before reaching a receiver site. Numerous estimate strategies have been investigated to remove the location inaccuracy brought on by multipath. Although multipath errors are a class of uncorrelated mistakes not eliminated by observation differentiation, they primarily restrict the performance of high-precision GPS receivers. The strategies for interference minimization are among the most crucial aspects of enhancing the efficiency of the GPS system.

The outliers are assessments that considerably differ from the majority of the observation sample. The irrelevancy of distinct instances and the emergence of uncertainty during the observation period are two of the main causes of inaccuracy for high-accuracy positioning systems. Regardless of the measures taken throughout the observation period and the improved models used to remove systematic errors, observations may still be affected by outliers. This often decreases the quality of the estimated states and makes them unreliable for any major interference. Accuracy can be increased by smoothing the range results to lessen the effects of NLOS propagation [3,4].

Further, the Kalman filter (KF) is a well-known estimator based on minimum mean-square error (MMSE). It is generally believed that the KF utilizes MMSE as the optimal criterion in linear systems with white Gaussian noise [3,4,5]. Non-Gaussian noises, on the other hand, are regularly present in many real-world environments and may significantly affect performance. Additionally, if non-Gaussian noise disrupts the system, the extended Kalman filter (EKF), an addition to the linear Kalman filter for addressing nonlinear systems, will become worse. The EKF does not use the high-order information of the process and measurement noises when the noise is non-Gaussian. To prevent divergence brought on by inaccurate modeling, the state vector was approximated using the innovation-based adaptive Kalman filter (AKF) technique [4,5,6]. Furthermore, the EKF struggles when the systems are mixed with colored noise, which happens in many practical contexts, as the estimated results are subject to substantial outliers and non-Gaussian noise. The particle filter (PF) demands a large investment in computer resources for continuous execution despite having the capability to handle non-Gaussian, nonlinear structures. The main disadvantage is the degree to which the EKF’s success relies upon a predetermined dynamic framework. To produce correct estimation findings, developers must be aware of the dynamic process and measurement models previously and assume that zero-mean Gaussian white noise corrupts both models. A procedure that adapts can also be used to dynamically spot modeling errors or system model uncertainty [7,8,9,10,11]. 

Moreover, when dealing with practical GPS navigation usage in non-Gaussian noise situations, algorithm robustness has emerged as a critical concern. The aforementioned innovative nonlinear filtering methods based on the entropy concept can be used as substitutes for GPS navigation processing. The mean-square error (MSE) can be used to build kernel adaptive filtering methods when additive noise in signal processing is assumed to follow a Gaussian process. Additionally, in information-theoretic learning (ITL), the concept of entropy is now widely used. The minimum error entropy (MEE) and maximum correntropy criteria (MCC) [12,13,14,15,16,17,18] have been used most frequently for resilient filter design when it comes to non-Gaussian systems. The approach ensures a satisfactory performance for non-Gaussian applications by suppressing the impacts of impulsive noise using kernel function in entropy. The statistical measure correntropy determines a connection between two or more variables by combining the probability density function (pdf) [19,20]. The accompanying MCC was applied as a new cost function for robust filtering to reduce the impact of non-Gaussian noise for vehicle navigation with multi-sensor information based on robust unscented Kalman filters. Furthermore, the robustness and adaptability for GNSS integration have been verified by using the cubature Kalman filter (CKF) [21,22,23,24]. The MCC was developed with the aid of an ITL technique and has been successfully used in a number of filter design applications, particularly when non-Gaussian noises are present.

Although, many studies have been carried out for the robust EKF known as the maximum correntropy criterion-based extended Kalman filter (MCCEKF), which were developed by substituting the MCC for the MSE condition in order to overcome the estimation issues in non-Gaussian noise conditions, particularly in the presence of impulse noise. However, relying on the relative entropy (also known as the Kullback–Leibler divergence) or minimax theory, a few other popular robust filter models are not studied widely. Therefore, the robust EKF with non-Gaussian noise known as MCCEKF works much better than the traditional EKF for the analysis of spectral redshift navigation based on the multipath information [25,26,27,28]. In this present study, it has been verified that MCC works well with M-estimators and is better suited for unknown noise and indeterminate features. The MCCEKF with a fixed adaptive kernel bandwidth (MCCEKF-AKB) has advantages due to its novel concept and computational simplicity. Also verified from the simulation results is a novel generic solution for the study of random structures with the generalized noise. In order to enhance the performance of EKF applications when non-Gaussian disturbances exist, MCCEKF uses the MCC rather than the MMSE as the optimization criterion for GNSS navigation processing. With the observed distance and its variance, regulated to eliminate the effects of colored Gaussian noise, the range error space may be restricted. The suggested novel MCCEKF-AKB has been shown to be more efficient at handling non-Gaussian data by adding an adaptable kernel bandwidth component to the MCCEKF.

The remainder of this paper is organized as follows. A brief review of the maximum correntropy criterion is reviewed in Section 2. In Section 3, the basic description of the MCC-based EKF (MCCEKF) is presented. In Section 4, the MCCEKF with adaptive kernel bandwidth (MCCEKF-AKB) is introduced. The proposed MCCEKF-AKB’s performance compared to the EKF and MCCEKF techniques are assessed using illustrative examples based on simulation experiments in Section 5. Finally, conclusions are given in Section 6.

## 2. Maximum Correntropy Criterion

Considering the autocorrelation of random phenomena, correntropy was first introduced to quantify consistency across delays. Later, it was expanded to evaluate the localized similarity of any two random variables. The correntropy between *X* and *Y* can be defined as follows:

(1) $$V_{\sigma }(X,Y)=E\left[\kappa_{\sigma }(X,Y)\right]=\int \int \kappa_{\sigma }(x,y)f_{XY}(x,y)dxdy$$

where E[⋅] represents the expectation function, the kernel function, and the joint probability density function (pdf) of X and Y are denoted as $\kappa_{\sigma }(X,Y)$, and $f_{XY}(x,y)$, respectively.

In general, the kernel size in terms of the Gaussian function can be expressed as

(2) $$\kappa_{\sigma }(x,y)=G_{\sigma }(x-y)=\exp\left(-\frac{{\left(x-y\right)}^{2}}{2\sigma^{2}}\right)$$

Generally, $f_{XY}(x,y)$ is calculated with a limited availability of data, which leads to computational difficulty. Therefore, the mean of a sample is used to compute the correntropy.

(3) $$V_{\sigma }\left(X,Y\right)=\frac{1}{N}\overset{N}{\underset{i=1}{\sum }}G_{\sigma }(e_{i})=\frac{1}{N}\sum_{i=1}^{N}G_{\sigma }\left(x_{i}-y_{i}\right)$$

where ${\left(x_{i},y_{i}\right)}_{i=1}^{N}$ are the N samples drawn from $f_{XY}(x,y)$.

After taking the Taylor series expansion of the Gaussian kernel function, we have

(4) $$V_{\sigma }\left(X,Y\right)=\overset{\infty }{\underset{n=0}{\sum }}\frac{{\left(-1\right)}^{n}}{2^{n}\sigma^{2n}n!}E\left[{\left(X-Y\right)}^{2n}\right]$$

In order to represent the high-order information within the data, the correntropy is a weighted sum for all the even-order moments of the variable (X−Y). Robust state estimation can be accomplished using correntropy as an optimization strategy because it measures if the degree of similarity exists between X and Y.

Although, several definitions were there, Renyi and Shannon have been created for diverse purposes since Shannon first proposed entropy in 1948 [29]. Correntropy, for instance, is frequently used in adaptive filtering and machine learning to determine the local similarity of random variables. The correntropy of two random variables that are presented in the kernel area is defined by information-theoretic and kernel techniques as a generalized local matching score. Several applications have been accepted by the MCC. For instance, machine learning, pattern recognition, and signal processing have all helped the MCC. The MCC and extended Kalman filters are introduced in this section to cope with non-white Gaussian noise, particularly the outliers. 

## 3. Extended Kalman Filter with Maximum Correntropy Criterion

Considering a model equation in the form of one discrete-time is

(5) $$x_{k}=f_{k-1}(x_{k-1})+w_{k-1}$$

(6) $$z_{k}=h_{k}(x_{k})+v_{k}$$

where $x_{k}\in {ℜ}^{n}$ and $w_{k}\in {ℜ}^{m}$ denote the nonlinear state vector and the process noise vectors, respectively, and $z_{k}\in {ℜ}^{m}$ and $v_{k}\in {ℜ}^{m}$ represent the measurement vector and the measurement noise vector, respectively. $Q_{k}$ is the process noise covariance matrix where $R_{k}$ is the measurement noise covariance matrix.

The vectors $w_{k}$ and $v_{k}$ in Equations (1) and (2) are zero-mean Gaussian white sequences having zero cross-correlation with each other:

$$E[w_{k}w_{i}^{T}]=Q_{k}\delta_{ik};\;E[v_{k}v_{i}^{T}]=R_{k}\delta_{ik};\;E[w_{k}v_{i}^{T}]=0\;\;for\;all\;i\;and\;k$$

where the superscript “T” denotes the matrix transpose. The symbol $\delta_{ik}$ stands for the Kronecker delta function given by

$$\delta_{ik}=\{\begin{array}{c} 1,\begin{array}{cc} & i=k \end{array}\\ 0,\begin{array}{cc} & i\neq k \end{array} \end{array}$$

The details algorithm for discrete-time EKF are as follows:

(1) Initialization of state vector ${\hat{x}}_{0}$ and state covariance matrix $P_{0}$.
(2) Predictions of the state vector and state covariance matrix are
  $${\hat{x}}_{k|k-1}=f_{k-1}({\hat{x}}_{k-1})$$
  $$P_{k|k-1}=\Phi_{k-1}P_{k-1}\Phi_{k-1}^{T}+Q_{k}$$
(3) Computation of the Kalman gain matrix: $K_{k}=P_{k|k-1}H_{k}^{T}{\left[H_{k}P_{k|k-1}H_{k}^{T}+R_{k}\right]}^{-1}$.
(4) Updating of the state vector: ${\hat{x}}_{k}={\hat{x}}_{k|k-1}+K_{k}[z_{k}-h_{k}({\hat{x}}_{k|k-1})]$.
(5) Updating of the error covariance: $P_{k}=\left[I-K_{k}H_{k}\right]P_{k|k-1}$.

where **I** is the unity matrix. For a linear dynamic system represented by $x_{k}=\Phi_{k-1}x_{k-1}+w_{k-1}$(for Equation (5)), the prediction of the state vector is then written as ${\hat{x}}_{k|k-1}=\Phi_{k-1}{\hat{x}}_{k-1}$ (for EKF algorithm step 2) and no Jacobian calculation is required. The relations between the linear approximation equation and the measurement matrices of the system can be expressed as

$$\Phi_{k-1}\approx {\frac{\partial f_{k}}{\partial x}|}_{x={\hat{x}}_{k-1}};\;H_{k}\approx {\frac{\partial h_{k}}{\partial x}|}_{x={\hat{x}}_{k|k-1}}$$

To obtain the filter gain $K_{k}$ for a Kalman filter, the performance index is minimized in terms of the MSE condition:

(7) $$J_{MSE}=tr(E[(x_{k}-{\hat{x}}_{k}){\left(x_{k}-{\hat{x}}_{k}\right)}^{T})=tr(P_{k})$$

where tr(⋅) denotes the trace of a matrix. In the absence of uncertainty in the process and measurement noise covariances, the performance index $J_{MSE}$ attains the global minimum by using the standard Kalman filter, taking the partial derivative of $P_{k}$ with respect to $K_{k}$, using $\frac{\partial [tr(P_{k})]}{\partial K_{k}}=0$ for a minimum.

Consider an augmented model given by state prediction error with the measurement equation as

(8) $$\left[\begin{array}{c} {\hat{x}}_{k|k-1}\\ z_{k} \end{array}\right]=\left[\begin{array}{c} x_{k}\\ h(x_{k}) \end{array}\right]+\left[\begin{array}{c} \delta x_{k}\\ v_{k} \end{array}\right]$$

where $\delta x_{k}$ is the state prediction error as $\delta x_{k}={\hat{x}}_{k|k-1}-x_{k}$ and we have

(9) $$\varphi_{k}=\left[\begin{array}{c} {\hat{x}}_{k|k-1}-x_{k}\\ v_{k} \end{array}\right]$$

(10) $$J_{\mathrm{MCC}}=G_{\sigma }(||z_{k}-h({\hat{x}}_{k|k-1})||)+G_{\sigma }(||{\hat{x}}_{k}-{\hat{x}}_{k|k-1}||)$$

The linear Kalman filter’s nonlinear variant can be obtained in detail from the linear model in [9] using the MCC as the base. Both of the two cost functions deal with various statistical parameters of the random variables. The cost function based on the MSE criterion is given by Equation (7), which is a function of the covariance matrix of the estimation error. On the other hand, the cost function based on the MCC given by Equation (11) is a function where the quantity involved is the innovation sequence $z_{k}-h({\hat{x}}_{k|k-1})$ weighted by $R_{{}_{k}}^{-1}$ and the residual ${\hat{x}}_{k}-{\hat{x}}_{k|k-1}$ weighted by $P_{k|k-1}^{-1}$. The weighted least squares (WLS) method can be used to create an alternate objective function and improve its robustness. Consequently, the objective purpose of MCC might be described as

(11) $$J_{\mathrm{MCC}}=G_{\sigma }(||z_{k}-h({\hat{x}}_{k|k-1})|{|}_{R_{k}^{-1}})+G_{\sigma }(||{\hat{x}}_{k}-{\hat{x}}_{k|k-1}|{|}_{P_{k|k-1}^{-1}})$$

where $\left|\right|\cdot {\left|\right|}_{A}$ represents the weighted norm of A. Taking the derivative of the objective function $J_{\mathrm{MCC}}$ with respect to ${\hat{x}}_{k}$ and setting its value to zero,

(12) $$\frac{\partial J_{\mathrm{MCC}}}{\partial {\hat{x}}_{k}}=0$$

(13) $$-\frac{1}{\sigma^{2}}G_{\sigma }(||z_{k}-h({\hat{x}}_{k|k-1})|{|}_{R_{k}^{-1}})H_{k}^{T}R_{k}^{-1}(z_{k}-h({\hat{x}}_{k|k-1}))+\frac{1}{\sigma^{2}}G_{\sigma }(||{\hat{x}}_{k}-{\hat{x}}_{k|k-1}|{|}_{P_{k|k-1}^{-1}})P_{k|k-1}^{-1}({\hat{x}}_{k}-{\hat{x}}_{k|k-1})=0$$

and the following equation can be obtained:

(14) $$P_{k|k-1}^{-1}{\hat{x}}_{k}-P_{k|k-1}^{-1}{\hat{x}}_{k|k-1}=\frac{G_{\sigma }(||z_{k}-h({\hat{x}}_{k|k-1})|{|}_{R_{k}^{-1}})}{G_{\sigma }(||{\hat{x}}_{k}-{\hat{x}}_{k|k-1}|{|}_{P_{k|k-1}^{-1}})}H_{k}^{T}R_{k}^{-1}(z_{k}-h({\hat{x}}_{k|k-1}))$$

which can be written as

(15) $$P_{k|k-1}^{-1}{\hat{x}}_{k}-P_{k|k-1}^{-1}{\hat{x}}_{k|k-1}=L_{k}H_{k}^{T}R_{k}^{-1}(z_{k}-h({\hat{x}}_{k|k-1}))$$

where

(16) $$L_{k}=\frac{G_{\sigma }(||z_{k}-h({\hat{x}}_{k|k-1})|{|}_{R_{k}^{-1}})}{G_{\sigma }(||{\hat{x}}_{k}-{\hat{x}}_{k|k-1}|{|}_{P_{k|k-1}^{-1}})}$$

Since $G_{\sigma }(||{\hat{x}}_{k}-{\hat{x}}_{k|k-1}|{|}_{P_{k|k-1}^{-1}})\approx G_{\sigma }(||{\hat{x}}_{k|k-1}-{\hat{x}}_{k|k-1}|{|}_{P_{k|k-1}^{-1}})=1$, we have

(17) $$L_{k}=G_{\sigma }\left(|\right|z_{k}-h({\hat{x}}_{k|k-1}){\left|\right|}_{R_{k}^{-1}})$$

and

(18) $$e_{k}=\left|\right|z_{k}-h({\hat{x}}_{k|k-1}){\left|\right|}_{R_{k}^{-1}}$$

We can write

$$L_{k}=\mathrm{diag}(G_{\sigma }\left(|\right|z_{k}-h({\hat{x}}_{k|k-1}{)||}_{R_{k}^{-1}}^{2}))=\mathrm{diag}(G_{\sigma }(e_{k,1}),\ldots ,G_{\sigma }(e_{k,m}))$$

then, finally, the factor can be obtained.

(19) $$L_{k}=\mathrm{diag}\left(\exp\left(-\frac{e_{k,1}^{2}}{2\sigma^{2}}\right),\ldots ,\exp\left(-\frac{e_{k,m}^{2}}{2\sigma^{2}}\right)\right)$$

The MCC based EKF results for the estimation with the updated gain can be written as

(20) $$K_{k}={\left(P_{k|k-1}^{-1}+L_{k}H_{k}^{T}R_{k}^{-1}H_{k}\right)}^{-1}L_{k}H_{k}^{T}R_{k}^{-1}$$

which can also be expressed as

(21) $$K_{k}=L_{k}P_{k|k-1}H_{k}^{T}{\left[L_{k}H_{k}P_{k|k-1}H_{k}^{T}+R_{k}\right]}^{-1}$$

(22) $${\hat{x}}_{k}={\hat{x}}_{k|k-1}+K_{k}\left[z_{k}-h({\hat{x}}_{k|k-1})\right]$$

(23) $$P_{k}=\left(I-K_{k}H_{k}\right)P_{k|k-1}$$

**Figure 1:**
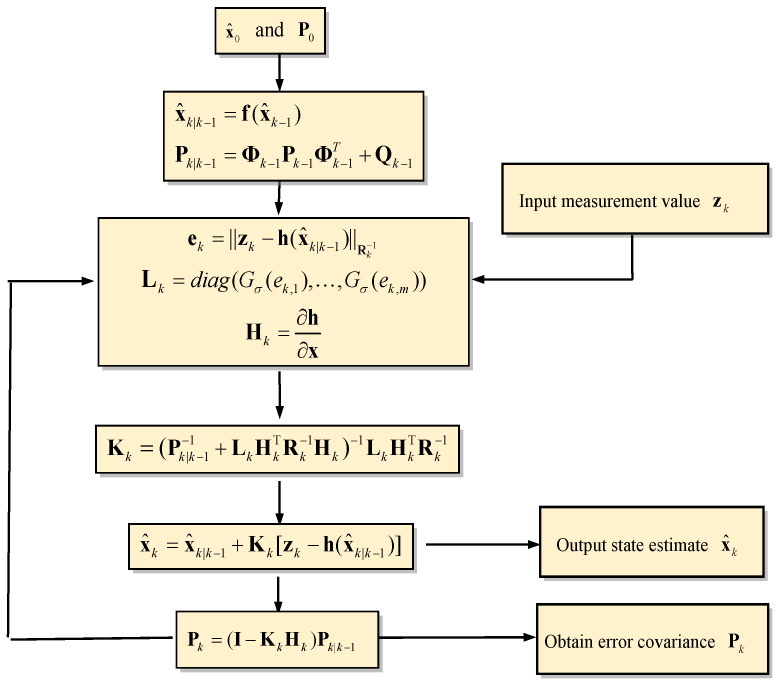
Flow diagram of the maximum correntropy criterion-based extended Kalman filter (MCCEKF) [19].

Figure 1 provides the flow chart for the maximum correntropy criterion-based extended Kalman filter (MCCEKF). The computation procedures are summarized as follows:

(1) Initialization of the state vector and state covariance matrix: ${\hat{x}}_{0}^{}$ and $P_{0}^{}$;
(2) Prediction of the state vector and the state covariance matrix: ${\hat{x}}_{k|k-1}$ and $P_{k|k-1}^{}$;
(3) Computation of the measurement innovation based on MCC to obtain the factor: $L_{k}$;
(4) Computation of the modified Kalman gain matrix: $K_{k}$;
(5) Updating the state vector and the error covariance: ${\hat{x}}_{k}$ and $P_{k}$;
(6) Repeating from Step (2) to evaluate the subsequent estimation cycle.

## 4. MCCEKF with Adaptive Kernel Bandwidth

Considering the initial measurement $z_{k}$ and the optimal prediction ${\hat{x}}_{k|k-1}$ acquired in the previous step, the innovations sequence is given by

(24) $$\upsilon_{k}=z_{k}-h({\hat{x}}_{k|k-1})$$

(25) $$\upsilon_{k}=z_{k}-{\hat{z}}_{k|k-1}^{}=h(x_{k})+v_{k}-{\hat{z}}_{k|k-1}^{}$$

The innovation shows the difference between the predicted measurement ${\hat{z}}_{k|k-1}^{}$ and the actual measurement $z_{k}$, while $z_{k}$ gives the extra data that the filter has access to as a new observation of the result. The weighted innovation, $K_{k}(z_{k}-{\hat{z}}_{k|k-1}^{})$, acts as a correction to the predicted estimation ${\hat{x}}_{k|k-1}^{}$ with the estimated ${\hat{x}}_{k}$. 

### 4.1. Innovation Information for Failure Detection and Adaptive Algorithms

Further, fetching both side variances, the obtained theoretical covariance, and the covariance matrix of the innovated sequence can be expressed as

(26) $$C_{\upsilon_{k}}=E[\upsilon_{k}\upsilon_{k}^{T}]=H_{k}P_{k|k-1}H_{k}^{T}+R_{k}$$

which can also be rewritten as

(27) $$C_{\upsilon_{k}}=H_{k}(\Phi_{k}P_{k}\Phi_{k}^{T}+\Gamma_{k}Q_{k}\Gamma_{k}^{T})H_{k}^{T}+R_{k}$$

Given that the length of the window is one and the degree of divergence (DOD) [15] is used as the record of the innovation covariance matrix,

(28) $$\xi =\upsilon_{k}^{T}\upsilon_{k}=tr(\upsilon_{k}\upsilon_{k}^{T})$$

This variable can be adjusted for the adaptive filtering or utilized to identify the divergence or outliers. Alternately, the concepts behind Equations (26) and (28) can be used to establish the parameters for determining the level of change in vehicle dynamics.

The other DOD measure, which is frequently employed as a straightforward test statistic for failure identification, is based on the ratio

(29) $$\mu =\frac{\upsilon_{k}^{T}\upsilon_{k}}{tr(C_{\upsilon_{k}})}$$

Similar to the above-mentioned parameters, the normalized innovation squared utilized in the case of fixed kernel bandwidth can be given by ${\left(\upsilon_{k}^{T}R_{k}^{-1}\upsilon_{k}\right)}^{1/2}$, where a factor β can be incorporated to obtain the extended version

(30) $$\beta \cdot {\left(\upsilon_{k}^{T}R_{k}^{-1}\upsilon_{k}\right)}^{1/2}$$

### 4.2. MCCEKF Based on Adaptive Kernel Bandwidth Mechanism

Employing these upper limits as an outline, the size of the kernel must be adjusted. Additionally, the kernel size is modified in the following way in the measurement-specific processing for outliers:

(31) $$\sigma_{j,k}=\lambda_{j,k}\cdot \sigma_{\max},\;\mathrm{for}\;j=1,2,\ldots ,m$$

where $\lambda_{j,k}$ denotes the adaptive factor for the jth measurement element at time step k, $z_{j,k}$, with the maximum kernel size $\sigma_{\max}$ = 150.

Further, $\lambda_{j,k}$ must be suitably adjusted for varying noise intensities. The correlation between the innovation factor and its covariance matrix $C_{\upsilon_{k}}$ is the first point to consider in finding the presence of measurement outliers, and $\alpha_{j,k}$ can be defined as

(32) $$\alpha_{j,k}=\frac{C_{\upsilon_{j,k}}}{\upsilon_{j,k}^{2}}$$

where $C_{\upsilon_{j,k}}$ is the *j*th diagonal element of the innovation covariance matrix $C_{\upsilon_{k}}=E[\upsilon_{k}\upsilon_{k}^{T}]=H_{k}P_{k|k-1}H_{k}^{T}+R_{k}$, with $\upsilon_{j,k}$ being the innovation term $\upsilon_{k}$ for the *j*th element, and $\upsilon_{j,k}=z_{j,k}-{\hat{z}}_{j,k|k-1}$. The $\lambda_{j,k}$ is obtained by using the relation

(33) $$\lambda_{j,k}=1-\exp(-\alpha_{j,k})$$

Again,

(34) $$\alpha_{j,k}=\frac{C_{\upsilon_{j,k}}}{\upsilon_{j,k}^{2}}=\frac{H_{k}P_{jj,k|k-1}H_{k}^{T}+R_{k}}{\upsilon_{j,k}^{2}}$$

Now, we have to find $\sigma_{j,k}$ based on the adaptive factor $\lambda_{j,k}$ according to $\alpha_{j,k}$.

(35) $$\sigma_{j,k}=\lambda_{j,k}\cdot \sigma_{\max},\;\mathrm{where}\;\lambda_{j,k}=1-\exp(-\alpha_{j,k})$$

**Figure 2:**
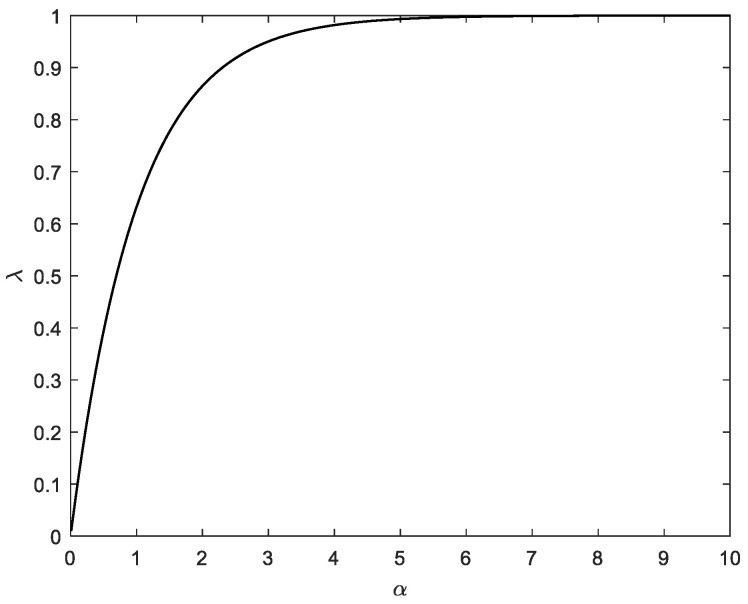
The values of adaptive factor as a function of kernel bandwidth.

**Figure 3:**
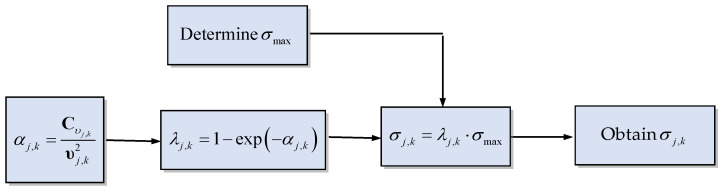
Flow chart for calculation of the kernel bandwidth.

The kernel size can be obtaining once the adaptive factor $\lambda_{j,k}$, is presented in Figure 2. Figure 3 shows the basic flow diagram to obtain the prefect kernel bandwidth. Further, the logic for the kernel bandwidth adapting algorithm is described in case studies.

Case 1: If <$\alpha_{j,k}>1$>, then <$\lambda_{j,k}$ is increased> (this indicates that if $C_{\upsilon_{k}}$ is larger than $\upsilon_{j,k}^{2}$, then the kernel bandwidth increases for maintaining the optimal performance).
Case 2: If <$\alpha_{j,k}<1$>, then <$\lambda_{j,k}$ is decreased> (this indicates that if $C_{\upsilon_{k}}$ is less than $\upsilon_{j,k}^{2}$, then the kernel bandwidth decreases for maintaining the robustness performance).

**Figure 4:**
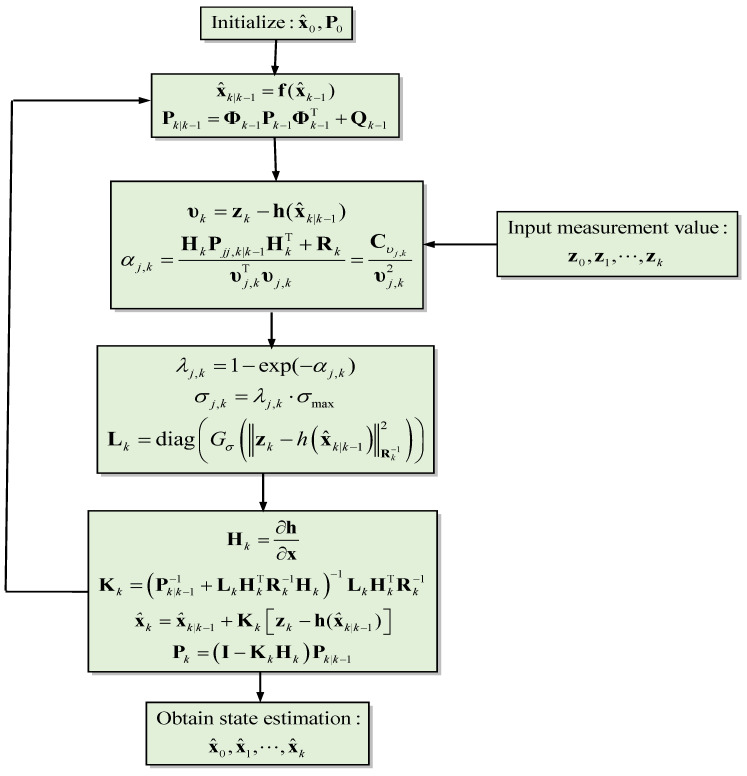
Flow chart of the MCCEKF-AKB.

The flow chart for the MCCEKF-AKB is presented in Figure 4, and the algorithm steps are summarized below:

(1) Initialization of the state vector and state covariance matrix: ${\hat{x}}_{0}^{}$ and $P_{0}^{}$;
(2) Prediction of the state vector and the state covariance matrix: ${\hat{x}}_{k|k-1}$ and $P_{k|k-1}^{}$;
(3) Obtaining $\sigma_{j,k}$ based on the adaptive factor $\lambda_{j,k}$ according to $\alpha_{j,k}$;
(4) Computation of the measurement innovation based on MCC to obtain the factor: $L_{k}$;
(5) Computation of the modified Kalman gain matrix: $K_{k}$;
(6) Updating the state vector and the error covariance: ${\hat{x}}_{k}$ and $P_{k}$;
(7) Repeating from Step (2) to perform the subsequent estimation cycle.

## 5. Results and Discussion

In order to demonstrate the efficiency of the suggested methodology for the context of a time-varying environment, computational simulations were carried out to assess the performance of the recommended strategy to traditional methods of GPS navigation processing. The authors designed the various navigation filters, such as the EKF, MCCEKF, and MCCEKF-AKB for the nonlinear systems, using MATLAB 2022^®^ codes and the commercially available programs Inertial Navigation System (INS) Toolbox [30] and Satellite Navigation (SatNav) Toolbox [31], both provided by GPSoft LLC, Athens, Ohio, USA. The proposed filtering algorithm does not affect the navigation signal since there is no information (e.g., velocity) aiding feedback to the tracking loop. The INS Toolbox is used for generating the vehicle’s trajectory, while the SatNav Toolbox is used for generating the satellite orbits and pseudoranges. 

**Figure 5:**
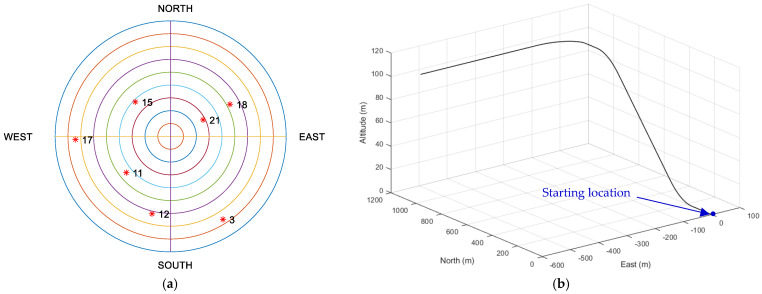
(**a**) The skyplot [19] and (**b**) the test trajectory for the simulated vehicle during the simulation interval.

The satellite skyplot for the seven GPS satellites (the red colored dot followed by the GPS ID number), and the test trajectory of the simulation are shown in Figure 5a,b, respectively. All the satellites are reachable, which is advantageous for ensuring proper navigation satellite geometry and accurate positioning results. The vehicle taken for simulation is assumed starting from the at 121.7775 degrees (east longitude) and 25.1492 degrees (north latitude) and is placed at a location with an altitude of 100 m, which is at ${\left[\begin{array}{ccc} -3042329.20 & 4911080.20 & 2694074.30 \end{array}\right]}^{T}$ m at the WGS-84 ECEF coordinate frame. The vehicle is the starting location and is considered as the (0,0,0) point in the local tangent East-North-Up (ENU) frame. 

The dynamic process of the GPS receiver is a linear one represented by the PV (Position–Velocity) model [3], where three position states, three velocity states, and two GPS receiver clock states (clock bias and drift) are involved, so that the state to be estimated is an 8×1 vector. The measurement model is a nonlinear system model, involved in the GPS pseudorange observables for n satellites

(36) $$\rho_{i}=\sqrt{{\left(x_{i}-x\right)}^{2}+{\left(y_{i}-y\right)}^{2}+{\left(z_{i}-z\right)}^{2}}+ct_{b}+v_{\rho_{i}},\;i=1,\ldots ,n$$

where (x,y,z) denotes the user position in three dimensions, $\left(x_{i},y_{i},z_{i}\right)$ denotes the i-th satellite’s position in three dimensions, c is the speed of light and $t_{b}$ is the receiver clock offset from system time, and $v_{\rho_{i}}$ is the pseudorange measurement noise.

In order to evaluate the performance when dealing with outlier-type multipath interferences, several additional and randomly produced errors are purposely introduced into the GPS pseudorange observed data while the vehicle is in motion. Since the errors caused by pseudorange observations indicate the accuracy of the measurement designs in the filters, we can concentrate on investigating these errors. The receiver thermal noise, multipath, ionospheric delay, and tropospheric delay are the error sources that affect the GPS outputs. The majority of inaccuracies are subsequently addressed by using the differential GPS (DGPS) mode, although the effects of multipath and the receiver’s thermal noise cannot completely be avoided in the current investigation. The error brought on by the thermal noise in the receiver appears as a Gaussian sequence with a mean of 0 m and a variance of 1 m^2^. For instance, using GNSS data to navigate in urban regions or environments with NLOS and multipath receptions is challenging. Additionally, the GPS sensor’s ability to locate itself precisely is hampered by the addition of incorrect data caused by these incidents. 

**Figure 6:**
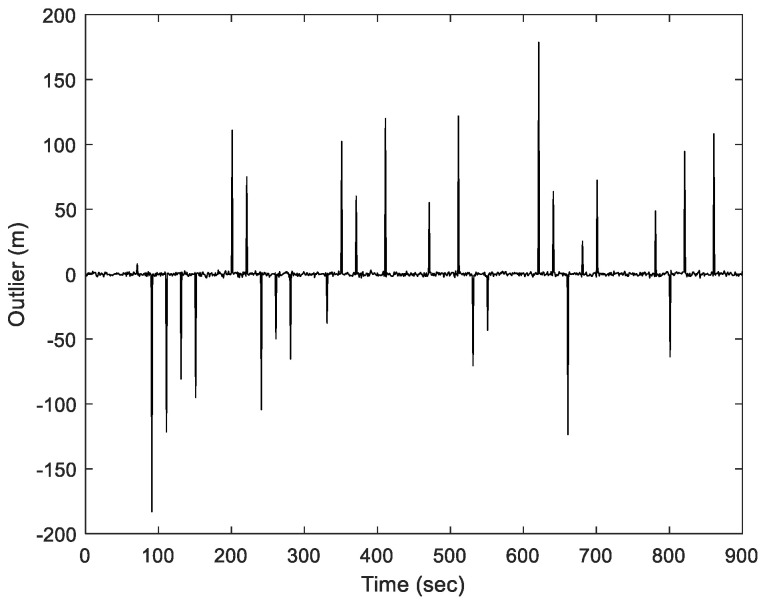
Information for the sequence involving outliers interference for Scenario 1.

The additional interference sequences due to the outliers for the pseudorange observations throughout the process are shown in Figure 6. There are 30 outliers in this simulation. During the coding process, these measurement errors could range from a few tens to several hundred meters.

### 5.1. Scenario 1: Pseudorange Observable Errors Based on Gaussian Mixture Distribution

Scenario 1 describes how to reduce outlier-type multipath problems that affect the pseudorange observable. Further randomly produced errors have been deliberately incorporated into the GPS pseudorange observational information over an aggregate of five time periods while the vehicle is traveling. 

The majority of the receiving-dependent errors are assumed to be avoided in this work using the differential GPS method, but receiver-independent problems like multipath and receiver measurement noise from thermal sources are not. We believe that the additional errors, such as multipath error and the thermal noise, have the Gaussian mixed distribution type, written in terms of the probability density function as follows:

(37) $$f_{X}(x)=\left(\frac{1-\varepsilon }{\sigma_{1}\sqrt{2\pi }}\right)\exp\left[-\left(\frac{x^{2}}{2\sigma_{1}^{2}}\right)\right]+\left(\frac{\varepsilon }{\sigma_{2}\sqrt{2\pi }}\right)\exp\left[-\left(\frac{x^{2}}{2\sigma_{2}^{2}}\right)\right]$$

which is equivalent with

(38) $$X\sim (1-\varepsilon )N(0,\;\sigma_{1}^{2})+\varepsilon N(0,\;\sigma_{2}^{2})$$

where $\sigma_{1}$ and $\sigma_{2}$ represent the standard deviations of the individual Gaussian distribution, and the error model contamination perturbing parameter is ε. To illustrate the viability of the suggested algorithm’s robustness in the form of non-Gaussian distribution, the concerned requirements are ε=1/30 (i.e., with approximate 3.33% contamination), and $\sigma_{2}=100\sigma_{1}=100\times 1$, which have been used to simulate the outlier type of multipath errors.

**Figure 7:**
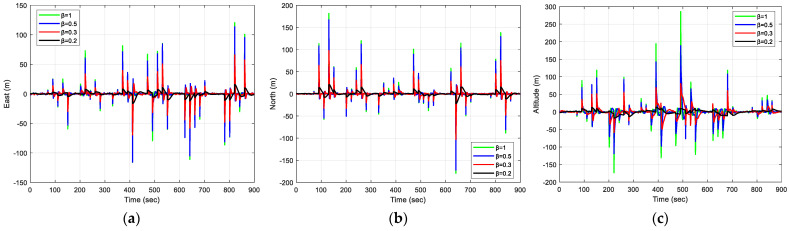
The results of position errors for various β values for Scenario 1: (**a**) East; (**b**) North; (**c**) Altitude.

**Figure 8:**
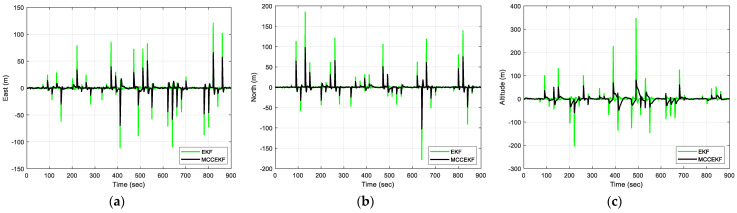
The results of position errors for EKF and MCCEKF for Scenario 1 with β = 0.3: (**a**) East; (**b**) North; (**c**) Altitude.

**Figure 9:**
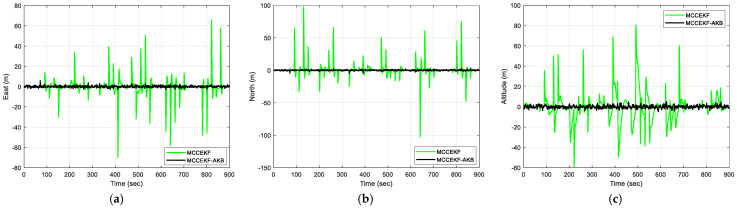
The results of position errors for MCCEKF and MCCEKF-AKB for Scenario 1 with β = 0.3: (**a**) East; (**b**) North; (**c**) Altitude.

**Figure 10:**
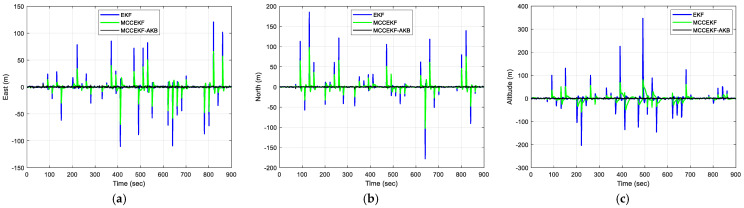
The results of position errors with three different techniques for Scenario 1 with β = 0.3: (**a**) East; (**b**) North; (**c**) Altitude.

The results of positioning errors for different techniques have been verified from Figure 7, Figure 8, Figure 9 and Figure 10. The position errors corresponding to different values of extended kernel bandwidth factor β with respect to the time (s) are presented in Figure 7. Further, in Figure 8, the positioning errors of the ENU frame for EKF and MCCEKF are shown. It can be noted that the convergence behavior of MCCEKF is significantly influenced by the kernel bandwidth, which can be fixed by the Banach Fixed-Point Theorem [7] in order to obtain a fixed-point convergence range. Furthermore, Figure 9 and Figure 10 show the different results for MCCEKF versus MCCEKF-AKB, and for the three approaches (EKF, MCCEKF, and MCCEKF-AKB), respectively. The results convey that the rate of convergence speed increases with adaptive kernel bandwidth. It will take a long time for the algorithm to diverge or converge if the kernel bandwidth is too tiny. 

**Figure 11:**
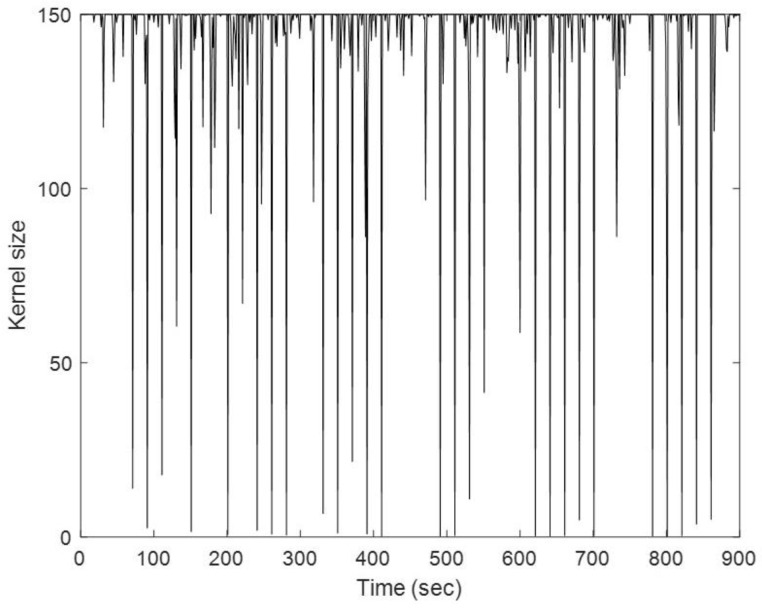
Time history of the kernel size for Scenario 1.

Faster convergence is guaranteed by a wider kernel bandwidth; however, this frequently results in a low performance in impulsive noise environments. The bandwidth can be manually adjusted or improved using trial-and-error techniques in real-world situations and it is not realistic in real-time implementations. Figure 11 shows the Monte Carlo trials for the kernel size against the time for considering the AKB for the MCCEKF, because the adaptive kernel size is related to the selection of $\sigma_{\max}$, it has resistance towards the outlier interferences [14].

### 5.2. Scenario 2: Pseudorange Observable Involving Outlier Type of Multipath Interferences with Time-Varying Variance in Measurement Noise

**Figure 12:**
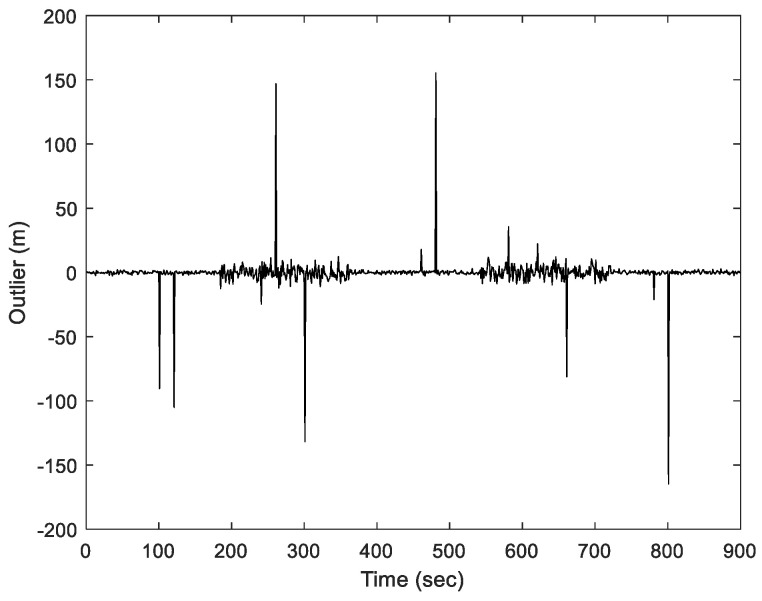
Information for the sequence involving outliers interference for Scenario 2.

**Figure 13:**
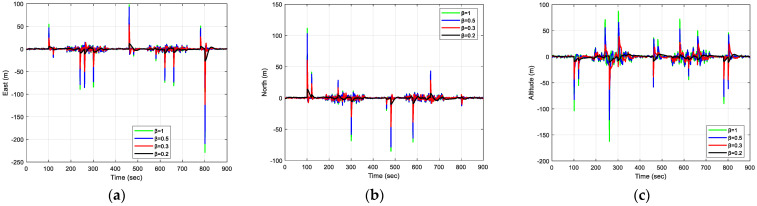
The results of position errors for various β values for Scenario 2: (**a**) East; (**b**) North; (**c**) Altitude.

**Figure 14:**
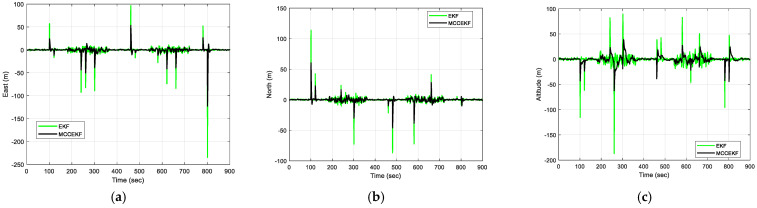
The results of position errors for EKF and MCCEKF for Scenario 2 with β = 0.3: (**a**) East; (**b**) North; (**c**) Altitude.

**Figure 15:**
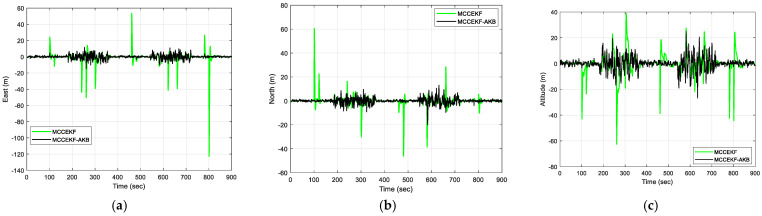
The results of position errors for MCCEKF and MCCEKF-AKB for Scenario 2 with β = 0.3: (**a**) East; (**b**) North; (**c**) Altitude.

**Figure 16:**
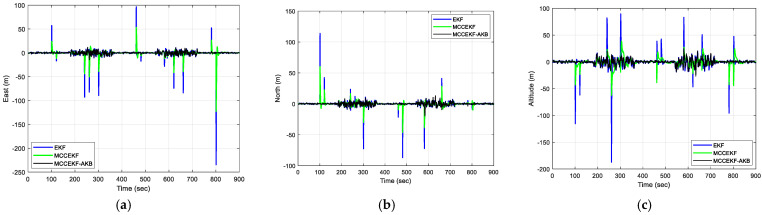
The results of position errors with three different techniques for Scenario 2 with β = 0.3: (**a**) East; (**b**) North; (**c**) Altitude.

For Scenario 2, we considered the pseudorange observable involving outlier type of multipath interferences with time-varying variance in the measurement noise. Figure 12 represents the information sequence affected due to the outlier interference, which indicates the variation in outliers with changing kernel size and time. For a more quantitative analysis, the results for various positioning errors for the ENU frames are represented in Figure 13, Figure 14, Figure 15 and Figure 16. The position errors corresponding to different values of extended kernel bandwidth factor β with respect to the time (s) are presented in Figure 13. Furthermore, Figure 14 represents the results for EKF and MCCEKF and Figure 15 shows the results for MCCEKF and MCCEKF-AKB, respectively, with a fixed value of kernel bandwidth. It is worth noting that the kernel bandwidth plays a vital role for the MCCEKF, which leads to the results of [9,11,28]. Additionally, the positioning errors among the three approaches are shown in Figure 16. The proposed algorithm for MCCEKF-AKB (Figure 4) achieves a good estimation of the results with a fixed kernel bandwidth size. For instance, it is observed that among all the plots of EKF, MCCEKF, MCCEKF-AKB techniques, and the MCCEKF-AKB achieve the desirable performance and better accuracy with a fixed kernel bandwidth. Moreover, the suggested approach for determining the maximum kernel size is more reliable and effective. When compared to previous robust filters, the filter often maintains a better and more consistent estimation accuracy within the fixed range [14,15,20,27], even though the choice of confidence level variables affects the position of outliers. Consequently, MCCEKF-AKB’s perfection and stability are verified.

**Figure 17:**
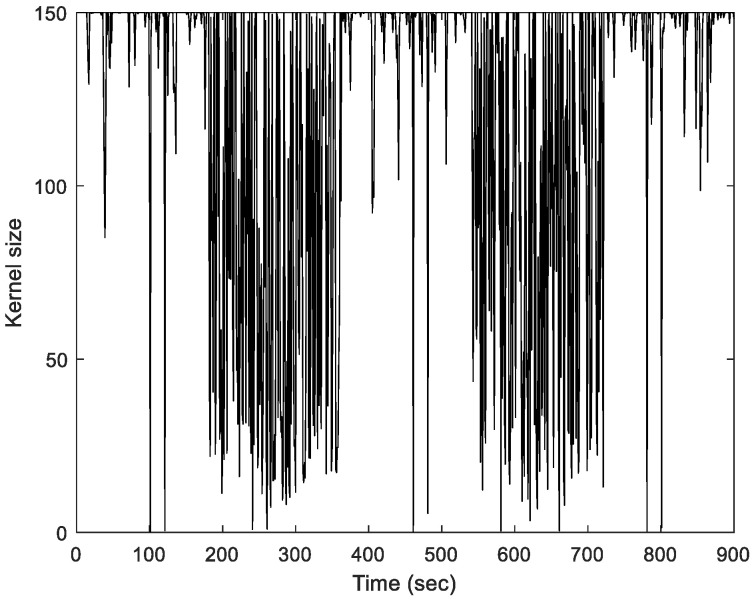
Time history of the kernel size for Scenario 2.

Finally, in Figure 17, the kernel size for the proposed study has been verified from the time history plot, which signifies the impact of kernel size. It can be seen that for both the Scenarios, MCCEKF-AKB shows qualitative performance in comparison to the other two methods, which also conveys the nature of the significant navigation preciseness efficiency. The aforementioned representations for kernel size in Figure 11 and Figure 17 indicate the improved performance that MCCEKF updates the posterior estimates under MCC using a fixed-point iterative approach. The initial value of the iteration can be set to the previous estimate $x_{k}$, and the tiny positive parameter offers a stop condition to acquire the convergent solution of the fixed-point iteration. It should be noted that when the kernel bandwidth, or σ, in the MCCEKF is greater than a particular threshold, the convergence of the fixed-point algorithm under MCC is assured. Generally speaking, an algorithm’s robustness and convergence speed increase with decreasing kernel bandwidth. However, the performance of the suggested algorithm will converge to that of the EKF algorithm as the kernel bandwidth increases, which verifies the consistent effectiveness of the navigation information processing [31].

## 6. Conclusions

In this paper, the algorithms for dealing with non-Gaussian or heavy-tailed errors, and outliers with multipath interference have been investigated by using three different filtering techniques. Since the multipath attenuation is crucial for improving positioning accuracy, a number of estimate techniques have been researched for high-precision positioning systems.

The MSE condition is used to create the standard EKF approach, which is constrained to the presumption that characterizes the linear and Gaussian ideal situation. Using an optimization strategy based on the correntropy criterion increases the resilience of nonlinear filters. A GPS navigation method based on MCC has been developed; it is constructive for interference errors with heavy tails and impulsive patterns. In addition, the behavior of the innovation-related variables has been implemented with the AKB for additional performance enhancement, which is helpful in constructing the MCCEKF-AKB. 

To demonstrate the effectiveness, simulations of GPS navigation tests have been performed for different filtering techniques. The adaptive filtering algorithm that employs the kernel bandwidth concept exhibits good potential as a substitute for the GPS navigation processor because it significantly improves navigation accuracy when compared to conventional methods, particularly the observations with non-Gaussian errors. For the purpose of illustration, two situations have been presented: (1) pseudorange observable errors based on Gaussian mixture distribution; and (2) pseudorange observable errors involved in the outlier type of multipath interferences with time-varying variance in measurement noise. The performance of other techniques, such as EKF, MCCEKF, and MCCEKF-AKB, have been compared, and the kernel bandwidth-based EKF algorithm, i.e., MCCEKF-AKB, has shown promising results in the enhancement of navigational accuracy.

## Data Availability

The data that support the findings of this study are available upon reasonable request from the authors.
